# Supplementary material for: α-Synuclein strain propagation is independent of cellular prion protein expression in a transgenic synucleinopathy mouse model
Source: PLoS Pathog. 2024 Sep 12;20(9):e1012517. doi: 10.1371/journal.ppat.1012517 (PMC11392418; doi:10.1371/journal.ppat.1012517)
Supplement: S1 Table — (PDF) [file ppat.1012517.s001.pdf]

S1 Table. List of mice with intercurrent illness removed from the study

Inoculum, route	Mouse genotype	Animal ID	Sex	Days post inoculation	Notes	Protease digestion pattern
S, IC	M83-*Prnp*$^{+/+}$	7790	F	93	Euthanized due to atypical illness	---
		7801	F	166	Euthanized due to atypical illness	---
		7802	M	100	Euthanized due to fight wounds	---
		7803	M	152	Found dead	Brain not collected
S, IC	M83-*Prnp*$^{0/0}$	7665	F	163	Euthanized due to atypical illness	---
		7681	F	166	Euthanized due to atypical illness	---
		6676	M	195	Euthanized due to fight wounds and urinary blockage	S
		6677	M	166	Found dead due to fighting injuries	Brain not collected
		6673	M	166	Found dead due to fighting injuries	Brain not collected
		7671	M	145	Euthanized due to atypical illness	---
NS, IC	M83-*Prnp*$^{+/+}$	7554	M	192	Euthanized due to fight wounds	NS
S, IP	M83-*Prnp*$^{+/+}$	9851	F	196	Euthanized due to atypical illness	---
		8868	M	156	Euthanized due to fight wounds	---
		8878	M	152	Found dead	---
		9858	M	231	Euthanized due to atypical illness	---
S, IP	M83-*Prnp*$^{0/0}$	9056	F	116	Found dead	Brain not collected
		8737	M	270	Euthanized due to atypical illness	---
NS, IP	M83-*Prnp*$^{+/+}$	8493	F	171	Euthanized due to atypical illness	---
		9394	F	370	Euthanized due to atypical illness	---
		8500	M	157	Found dead	Brain not collected
		8502	M	168	Found dead	Brain not collected
NS, IP	M83-*Prnp*$^{0/0}$	8740	F	375	Euthanized due to atypical illness	---
		8482	M	192	Euthanized due to atypical illness	---
		8484	M	227	Euthanized due to atypical illness	---
		8485	M	227	Euthanized due to atypical illness	---
		8731	M	109	Euthanized due to atypical illness	---
		8739	M	160	Found dead	Brain not collected

Three dashes (---): Brains collected and analyzed by protease digestion assay; no protease-resistant α-syn detected by immunoblotting.
