# Supplementary figures and images for: α-Synuclein strain propagation is independent of cellular prion protein expression in a transgenic synucleinopathy mouse model
Source: PLoS Pathog. 2024 Sep 12;20(9):e1012517. doi: 10.1371/journal.ppat.1012517 (PMC11392418; doi:10.1371/journal.ppat.1012517)

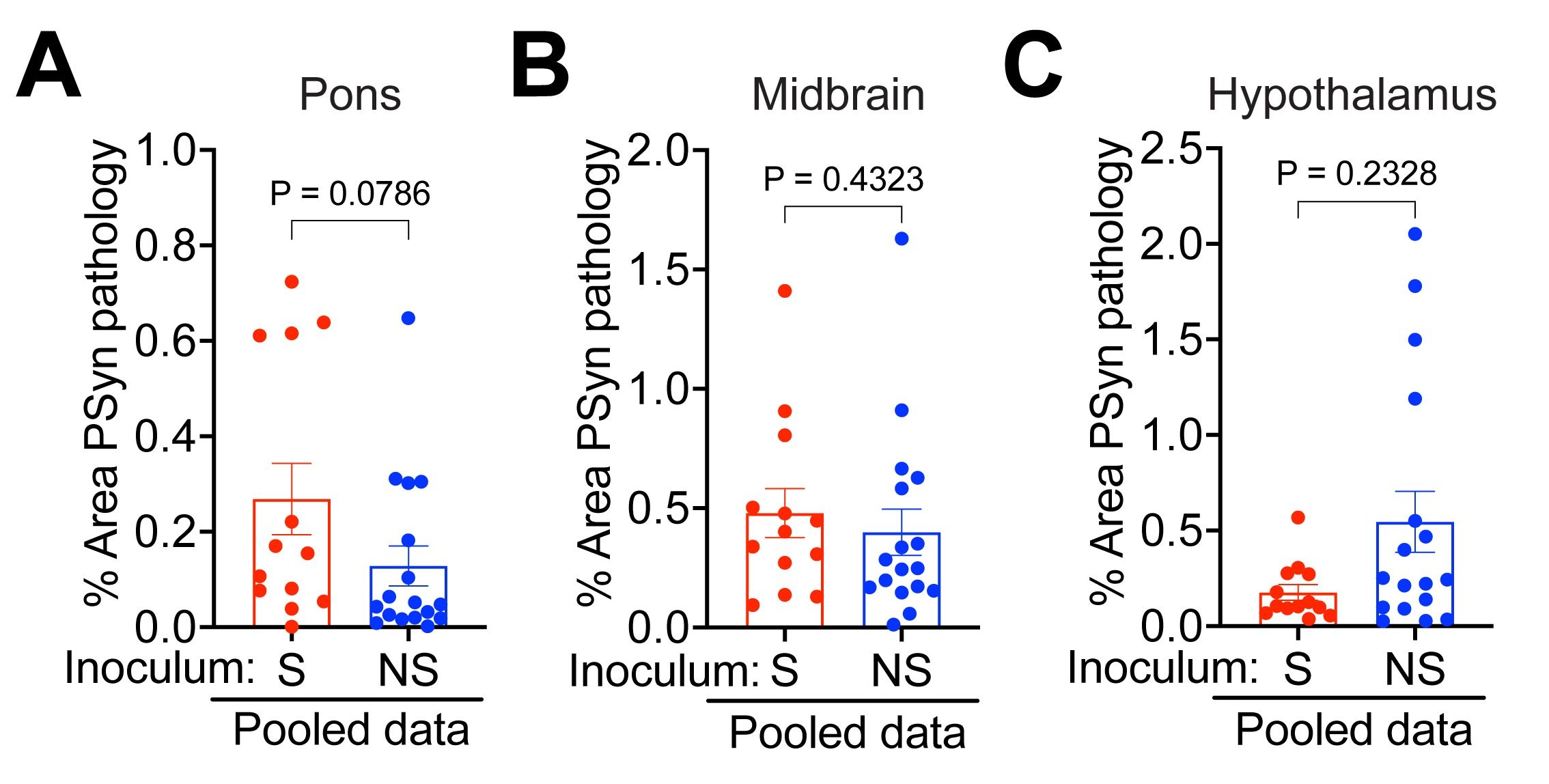

Supplement: S1 Fig — Pooled quantitative data for the area covered by PSyn staining in the pons (A), midbrain (B), and hypothalamus (C) of symptomatic M83-Prnp+/+ and M83-Prnp0/0 mice inoculated intracerebrally with either S (n = 13) or NS (n = 17) strain. The graphs display mean ± s.e.m. Statistical significance was assessed using a Mann-Whitney test. (TIF) [file ppat.1012517.s002.tif]

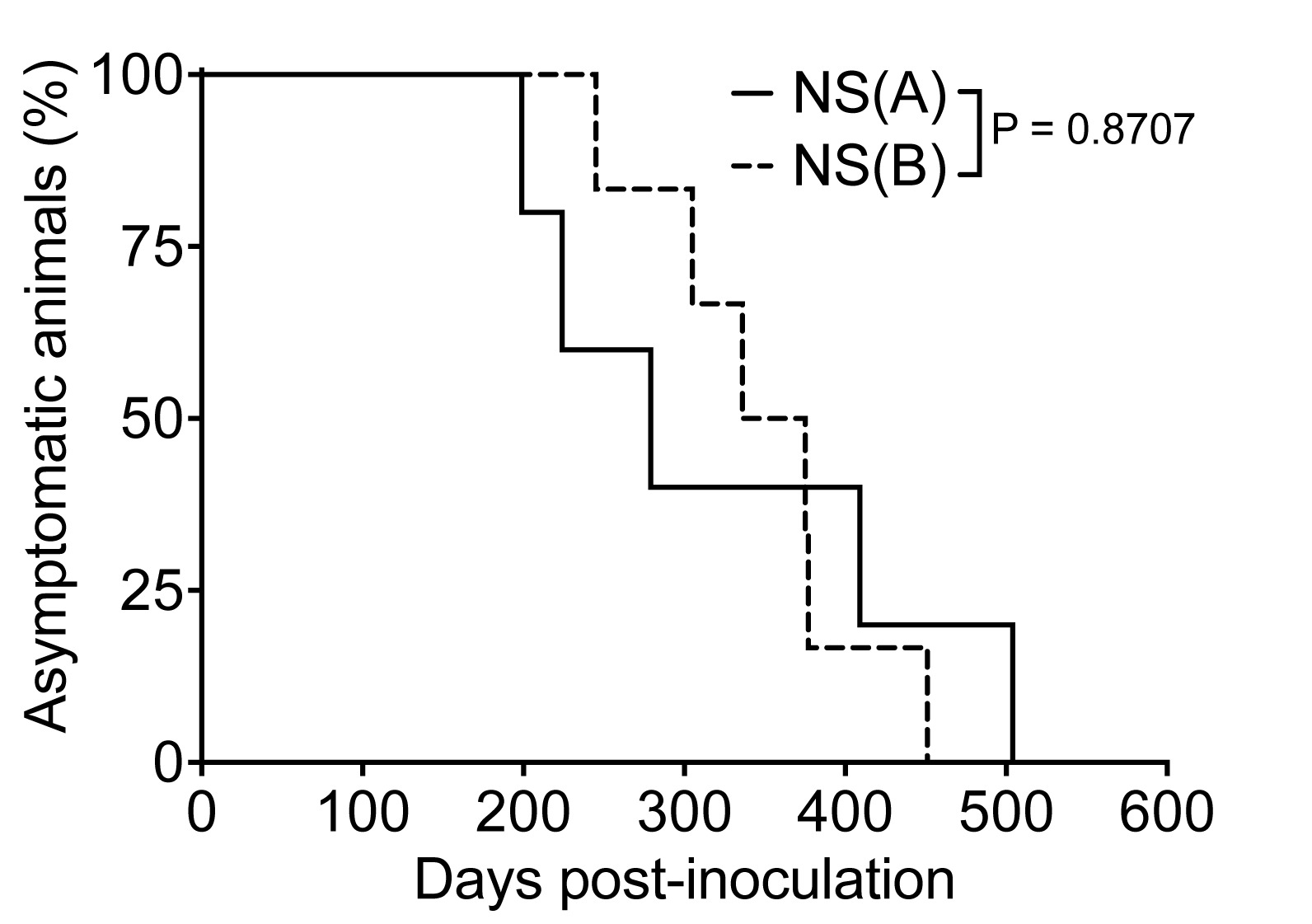

Supplement: S2 Fig — Kaplan-Meier survival curves for M83-Prnp+/+ and M83-Prnp0/0 mice (pooled data) inoculated intraperitoneally with the NS strain. Based on patterns of protease-resistant α-syn, mice were classified as either subtype A (n = 5) or B (n = 6). Statistical significance was assessed using the Log-rank test. (TIF) [file ppat.1012517.s003.tif]

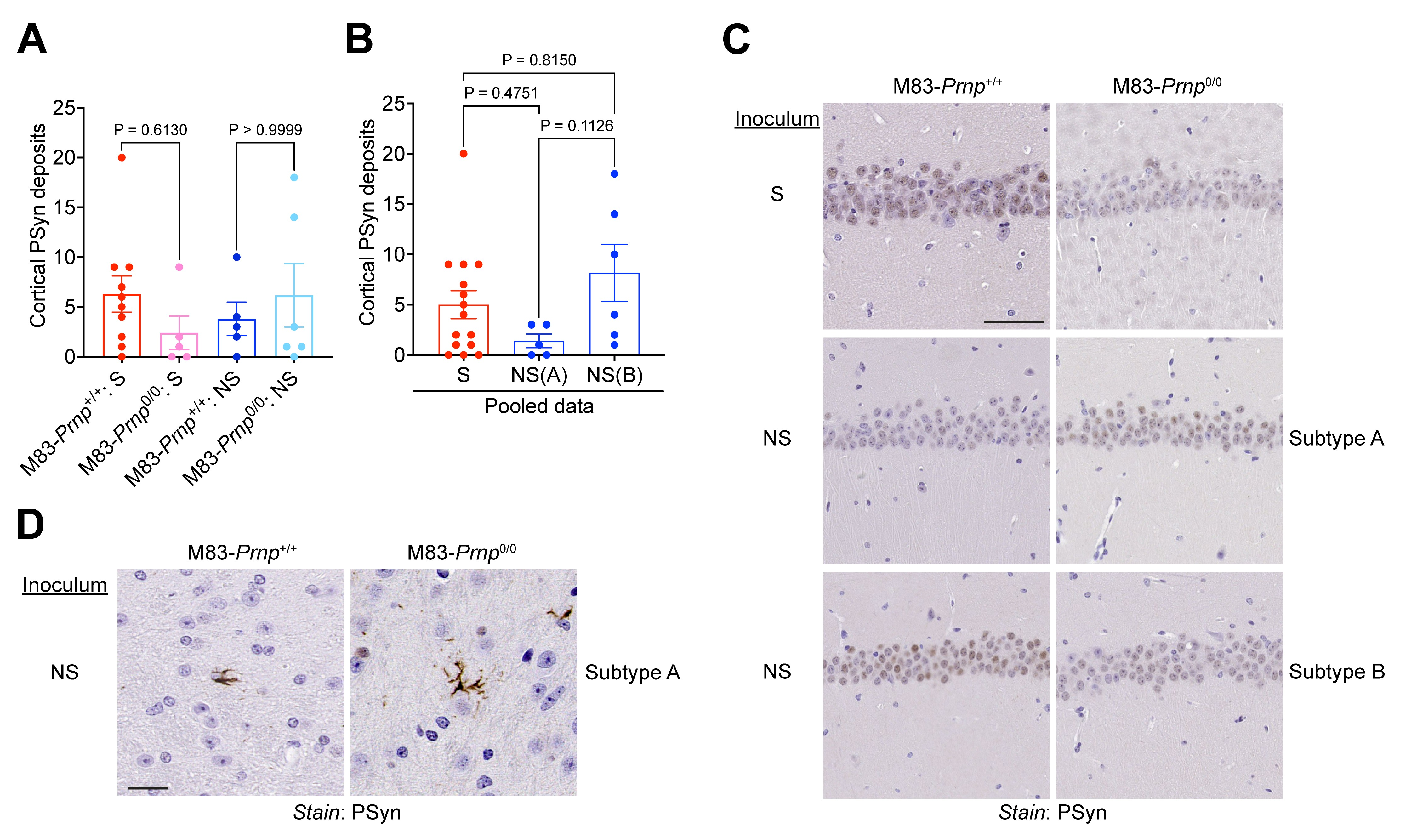

Supplement: S3 Fig — A) Quantification of the number of PSyn-positive neurons in the cortex of symptomatic M83-Prnp+/+ and M83-Prnp0/0 mice inoculated intraperitoneally with the S or NS strain (n = 5–10 mice per experimental condition). B) Pooled PSyn-positive neuronal counts in the cortex of M83-Prnp+/+ and M83-Prnp0/0 mice inoculated intraperitoneally with S strain (n = 15) or exhibiting the subtype A (n = 5) or subtype B (n = 6) phenotype following intraperitoneal inoculation with the NS strain. The graphs in panels A and B display mean ± s.e.m., and statistical significance was assessed using a Kruskal-Wallis test followed by Dunn’s multiple comparisons test. C) Representative images of PSyn-stained sections from the hippocampal CA1 region of symptomatic M83-Prnp+/+ and M83-Prnp0/0 mice inoculated intraperitoneally with the S or NS strains. Scale bar = 50 μm. D) Representative images of PSyn-stained sections from the thalamus of symptomatic M83-Prnp+/+ and M83-Prnp0/0 mice inoculated intraperitoneally with the NS strain and exhibiting the subtype A phenotype. Scale bar = 20 μm. (TIF) [file ppat.1012517.s004.tif]

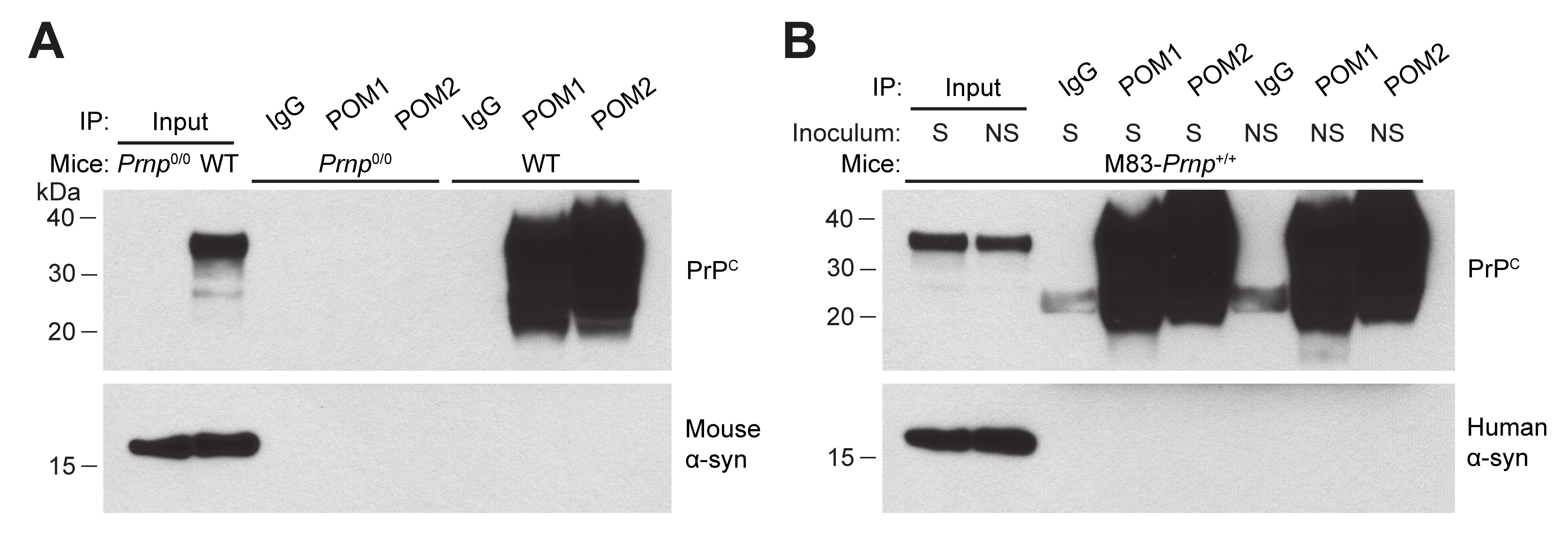

Supplement: S4 Fig — A) Immunoblots for PrPC and mouse α-syn following immunoprecipitation of PrPC from 500 μg of detergent-extracted brain homogenates from healthy non-transgenic wild-type (WT) and Prnp0/0 mice. PrPC was immunoprecipitated using either the POM1 or POM2 antibodies. A non-specific mouse IgG was used as a control. Input lanes contain 20 μg of detergent-extracted brain homogenate. PrPC was detected using the antibody HuM-D18 and mouse α-syn was detected using D37A6. B) Immunoblots for PrPC and human α-syn following immunoprecipitation of PrPC from 500 μg of detergent-extracted brain homogenates from symptomatic M83-Prnp+/+ mice inoculated intracerebrally with either S or NS strain. PrPC was immunoprecipitated using either the POM1 or POM2 antibodies. A non-specific mouse IgG was used as a control. Input lanes contain 10 μg of detergent-extracted brain homogenate. PrPC was detected using the antibody HuM-D18 and human α-syn was detected using MJFR1. (TIF) [file ppat.1012517.s005.tif]
